# Schrödinger’s microbes: Tools for distinguishing the living from the dead in microbial ecosystems

**DOI:** 10.1186/s40168-017-0285-3

**Published:** 2017-08-16

**Authors:** Joanne B. Emerson, Rachel I. Adams, Clarisse M. Betancourt Román, Brandon Brooks, David A. Coil, Katherine Dahlhausen, Holly H. Ganz, Erica M. Hartmann, Tiffany Hsu, Nicholas B. Justice, Ivan G. Paulino-Lima, Julia C. Luongo, Despoina S. Lymperopoulou, Cinta Gomez-Silvan, Brooke Rothschild-Mancinelli, Melike Balk, Curtis Huttenhower, Andreas Nocker, Parag Vaishampayan, Lynn J. Rothschild

**Affiliations:** 1 Department of Microbiology, The Ohio State University, 484 West 12th Avenue, Columbus, OH 43210 USA; 2 Department of Plant & Microbial Biology, University of California, Berkeley, 111 Koshland Hall, Berkeley, CA 94720 USA; 3 Biology and the Built Environment Center, Institute of Ecology and Evolution, University of Oregon, Eugene, OR 97403 USA; 4 Institute of Ecology and Evolution, University of Oregon, Eugene, OR 97403 USA; 5 Department of Earth and Planetary Sciences, University of California, Berkeley, Berkeley, CA 94720 USA; 6 Genome Center, University of California Davis, 451 Health Sciences Drive, Davis, CA 95616 USA; 7 Department of Civil and Environmental Engineering, Northwestern University, 2145 Sheridan Road, Evanston, IL 60208 USA; 8 Department of Biostatistics, Harvard T.H. Chan School of Public Health, 665 Huntington Avenue, Boston, MA 02115 USA; 9 The Broad Institute of MIT and Harvard, 415 Main Street, Cambridge, MA 02142 USA; 10 Lawrence Berkeley National Lab, 1 Cyclotron Road, 955-512L, Berkeley, CA 94720 USA; 11 Universities Space Research Association, NASA Ames Research Center, Mail Stop 239-20, Building 239, room 377, Moffett Field, CA 94035-1000 USA; 12 Department of Mechanical Engineering, University of Colorado at Boulder, 1111 Engineering Drive, 427 UCB, Boulder, CO 80309 USA; 13 Department of Environmental Science, Policy, and Management, University of California, Berkeley, CA 94702 USA; 14 Division of Biological Sciences, The University of Edinburgh, Mayfield Road, Edinburgh, EH9 3JH UK; 15 Department of Earth Sciences – Petrology, Faculty of Geosciences, Utrecht University, P.O. Box 80.021, 3508 TA Utrecht, The Netherlands; 16 IWW Water Centre, Moritzstrasse 26, 45476 Mülheim an der Ruhr, Germany; 17 Biotechnology and Planetary Protection Group, Jet Propulsion Laboratory, California Institute of Technology, Pasadena, CA USA; 18 Planetary Sciences and Astrobiology, NASA Ames Research Center, Mail Stop 239-20, Building 239, room 361, Moffett Field, CA 94035-1000 USA; 19 Current Address: Department of Plant Pathology, University of California, Davis, CA USA

**Keywords:** DNA sequencing, Flow cytometry, Infectivity, Live/dead, Low biomass, Metagenomics, Microbial ecology, PMA, RNA, qPCR, Viability

## Abstract

While often obvious for macroscopic organisms, determining whether a microbe is dead or alive is fraught with complications. Fields such as microbial ecology, environmental health, and medical microbiology each determine how best to assess which members of the microbial community are alive, according to their respective scientific and/or regulatory needs. Many of these fields have gone from studying communities on a bulk level to the fine-scale resolution of microbial populations within consortia. For example, advances in nucleic acid sequencing technologies and downstream bioinformatic analyses have allowed for high-resolution insight into microbial community composition and metabolic potential, yet we know very little about whether such community DNA sequences represent viable microorganisms. In this review, we describe a number of techniques, from microscopy- to molecular-based, that have been used to test for viability (live/dead determination) and/or activity in various contexts, including newer techniques that are compatible with or complementary to downstream nucleic acid sequencing. We describe the compatibility of these viability assessments with high-throughput quantification techniques, including flow cytometry and quantitative PCR (qPCR). Although bacterial viability-linked community characterizations are now feasible in many environments and thus are the focus of this critical review, further methods development is needed for complex environmental samples and to more fully capture the diversity of microbes (e.g., eukaryotic microbes and viruses) and metabolic states (e.g., spores) of microbes in natural environments.

## Background

In the classic Monty Python “Dead Parrot” comedy sketch, John Cleese plays an irate pet store customer, who complains to the shopkeeper that he was sold a dead bird. While the shopkeeper insists that the bird is “only resting,” the customer bangs his wooden-stiff bird on the counter, screaming, “Hello, Polly” into its ear with no response. It is quite clear that the bird is dead. Distinguishing living from dead microbes is seldom so obvious, but it can have important and even lethal consequences if, for example, living pathogenic microbes are in pharmaceuticals, food, or swimming pools. There are huge environmental implications if a toxic algal bloom is alive, dead, or dying, and medical consequences may depend on the number and distribution of live or dead cells in microbial biofilms on heart valves or teeth. The structure and function of microbiomes depends on which members of the community are alive or dead. The possibility of finding life beyond Earth relies utterly on the sterility of spacecraft and their payloads.

As with Erwin Schrödinger’s quantum mechanics thought experiment, in which a cat could appear to be simultaneously both alive and dead until a measurement is made, a dedicated assessment of living and/or dead microorganisms is usually a requirement to know whether members of microbial communities are alive or dead. The methods used for live/dead determinations and assessments of microbial activity can affect conclusions about both living and dead microorganisms in consortia. Here, we review and, in the process, identify gaps in currently available techniques to distinguish between living and dead microbes.

The question of whether microbes are alive or dead began with the birth of the field of microbiology in 1683 when Anton van Leeuwenhoek recorded the first observation of bacteria. Nearly 200 years later, Robert Koch defined a pure culture and colony [1], allowing for quantitative estimates of the number of viable microorganisms in bacterial samples. Soon afterwards, agar was used in research and the Petri dish was developed, paving the way for standardized bacterial observations. These tools allowed for standards for the determination of microbiological viability: the ability to culture microbial cells. The cultivation-based viability assay shows an observable division of a single cell into colonies on agar plates or in liquid medium, thereby proving that the cells are alive (reviewed in [2]). This method is still the “gold standard” for a variety of applications today. For example, in an effort to check the sterility of their clean rooms and spacecraft, the National Aeronautics and Space Administration (NASA) incubates strips from each room on agar plates and assesses growth [3], although alternatives to agar have also been used on the Russian space station MIR to avoid contamination [4]. A similar method is used to detect bacteria when performing water quality testing by culturing water samples on agar plates [5], and these techniques are routinely used for testing and regulatory compliance in hospitals, the pharmaceutical industry, other medical fields, food protection, and the cosmetics industry [6–11]. While individuals from these fields may find some aspects of this review useful, microbial ecologists working with environmental samples from a variety of ecosystems are the intended audience.

Since the beginning of microbiology, culture-based methods have been used to assess viability. However, culture-independent assessments of microbial consortia, particularly through DNA sequencing, have allowed for the resolution of microbial community structure and function with a level of detail unimaginable a decade or so ago. Unfortunately, culture-independent DNA sequencing methods cannot unequivocally differentiate between living and dead cells. DNA can persist in the environment, resulting in extracellular DNA and DNA from dead cells that is indistinguishable from DNA representing living cells [12–15]. DNA and/or cellular material from dead microorganisms may be important in certain contexts, for example as bioavailable nutrients, sources of genetic material, historical representations of past organisms or ecological conditions, and/or as agents of respiratory ailments [14, 16–19]. However, it is the live microorganisms that have the potential to grow in, adapt to, and actively change a given environment. Without the selective identification of the living microbes, counting techniques and DNA sequencing approaches are likely to overestimate the types and numbers of viable taxa and/or active metabolic processes in microbial communities [15]. This is problematic not only for comparative microbial ecology but also for pathogen detection, cleanliness estimations, bioburden analysis, and antibiotic susceptibility testing [20]. For example, should a public beach be closed strictly based on DNA sequencing-based determination of contamination without, for example, microscopy, growth, or toxin testing?

The delineation between life and death is complex and debatable, and detailed considerations on the meaning of life and death in microbiology can be found elsewhere (e.g., [21]). In short, it is generally accepted (but not a universal rule) that a cell must be intact, capable of reproduction, and metabolically active, in order to be considered alive, and different viability assessments are designed to measure one or more of these properties, either directly or by proxy. These so-called live/dead protocols typically address one of the three aspects of microbial viability: (1) the existence of an intact, functional cell membrane, (2) the presence of cellular metabolism or energy, or (3) the possession of self-replicating DNA that can be transcribed into RNA, which, if applicable, can subsequently be translated into protein (adapted from [22]). Viruses, while not technically “alive” per these (and many) definitions, can be infectious or inactivated, and distinguishing between the two states can be more difficult than distinguishing living and dead forms of other microorganisms.

**Fig. 1:**
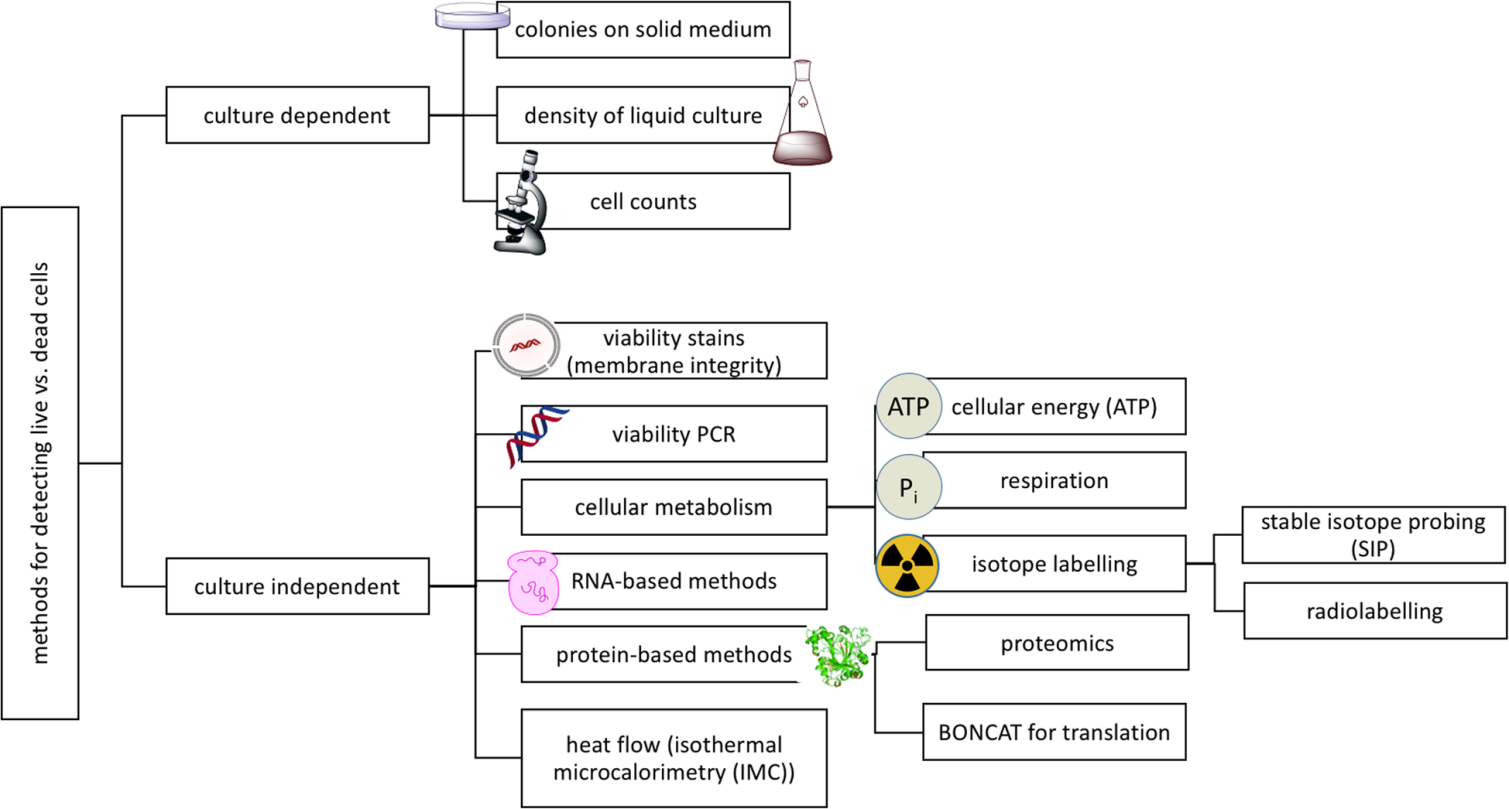
Overview of techniques to distinguish live from dead microbes. Both culture-dependent and culture-independent methods offer a variety of approaches, examples of which are categorized here, with culture-independent methods described further in the text

While we provide background for a variety of techniques, we focus on those that are compatible with microbial ecological studies that use nucleic acid sequencing approaches, because of their widespread utility. Specifically, we assess the applicability of these techniques to diverse microbial taxa and life stages while focusing on prokaryotes, diverse sample types (including non-aqueous and/or low-biomass samples), and compatibility with downstream analytical approaches, particularly next-generation sequencing (e.g., metagenomics, metatranscriptomics, and/or targeted amplicon/marker gene sequencing) and high-throughput counting techniques. We review the most commonly used, cutting-edge techniques, including viability PCR and RNA sequencing approaches and their compatibility with flow cytometry, quantitative PCR (qPCR), and digital PCR, which may be useful for quantifying viable populations in microbial ecological studies. We then consider other techniques for viability and activity assessments, including measurements of cellular energy (adenosine 5′-triphosphate (ATP)), metaproteomics, isotope probing, measurements of membrane potential and respiratory activity, and measurements of heat flow. Additional live/dead techniques, including many dyes and stains, are reviewed elsewhere [22–24]. Viability assessment techniques and compatible sample types, microbial taxa, and downstream analytical techniques are shown diagrammatically in Fig. 1, and their relative properties are summarized in Table 1.

**Table 1:** Comparison of commonly used techniques to identify living and/or dead cells, particularly those applicable or potentially applicable to microbial communities

Method	Approach	Compatible with next-generation sequencing?	Compatible techniques	Compatible sample types	Applicable to low-biomass samples?	Compatible biological entities	Pros	Cons	References
Cultivation	Plating and/or liquid culture to visualize actively multiplying cells	Y	Many	Many (nearly all environments)	Y	Many (some representatives across broad phylogenetic groups of bacteria, archaea, fungi, spores, and viruses have been cultured)	Unambiguous detection of viable microbes when cultivable	Many microbes are not (yet) cultivable, therefore not practical for characterizing the viable portion of most microbial communities	[33, 34]
Propidium iodide (PI)	Dye binding to DNA in membrane-compromised cells and extracellular DNA; sometimes used in combination with total nucleic acid stains	N^a^	Many, e.g., epifluorescence microscopy, confocal laser scanning microscopy, flow cytometry, fluorometry	Many (e.g., marine, freshwater, air, and soil samples), but samples must be in aqueous solution	N	Many (e.g., demonstrated for some psychrophilic, halophilic, and methanogenic archaea and some yeast, fungi, Gram + and Gram − bacteria)	Absolute live/dead abundance quantification is possible when combined with dyes that can permeate intact membranes; readily available in commercial kits	Known to stain viable cells of some species, and some organisms may not stain properly	[52, 47]
Propidium monoazide (PMA)	Dye binding to DNA in membrane-compromised cells and extracellular DNA	Y	Many, e.g., PCR, qPCR, MDA metagenomics, FISH, LAMP, microarrays, DGGE	Many (e.g., marine, clean room, sediment, soil, biofilm, and wastewater treatment samples), but samples must be in aqueous solution	Y	Many (e.g., demonstrated for some methanogenic archaea, some Gram + and Gram − bacteria, some viruses, and some spores)	Easy to perform and relatively fast; compared to EMA, more selective and less cytotoxic; several options for protocol trouble-shooting (see text)	Optimization of the method might be necessary; known to stain viable cells of some species and not stain dead cells of other species (but generally more selective in this regard than EMA)	[81, 73, 74, 75, 66, 69, 71, 70, 20]
Ethidium monoazide (EMA)	Dye binding to DNA in membrane-compromised cells and extracellular DNA	Y	Flow cytometry and PCR	Many (e.g., pure cultures from marine and food samples; likely similar to PMA, but not widely tested), but samples must be in aqueous solution	N^a^	Less well studied, but likely similar to PMA above	Several options for protocol troubleshooting (see text)	Known to stain viable cells of some species; less selective and more cytotoxic than PMA	[60, 57, 58, 59]
Alexa Fluor Hydrazide (AFH)	Dye binding to aldehydes and ketones in polysaccharides, glycoproteins, and/or in irreversibly damaged proteins (penetrates membrane-compromised cells)	N	Cultivation, flow cytometry, microscopy	Unknown	Unknown	Only tested on eukaryotic cells and a few bacteria (e.g., *E. coli*) so far	Low false-positive rate; does not require the presence of nucleic acids for staining; the ability to stain dead cells increases with cell age (as opposed to some nucleic acid stains)	Has not be applied at the community scale	[182]
RNA analyses (e.g., metatranscriptomics)	Quantifying or sequencing mRNA and/or rRNA	Y	MVT (for pre-rRNA), qPCR, PCR, RNA sequencing	Any, given sufficient RNA yield and quality	Y (rRNA), N (mRNA)	Many (e.g., archaea, Gram + bacteria, Gram − bacteria, fungi, spores if RNA can be extracted, actively replicating viruses, and RNA viruses)	Can reveal phylogeny and metabolic potential (mRNA) of likely viable and/or recently active microbes	mRNA has short half-life; rRNA is present in dormant cells; the extraction of high-quality RNA can be challenging	[103, 105, 116, 115]
Cellular energy measurements	Measuring ATP concentration	N	Flow cytometry, epifluorescence microscopy, CCD camera	Many (e.g., marine, built environment, food, bioaerosols, and clean room samples)	Y	Many (e.g., archaea, Gram + bacteria, Gram − bacteria, and fungi)	ATP concentration has high correlation with number of metabolically active cells; rapid and affordable assay	Can overestimate ATP concentrations because of extracellular ATP; metabolically dormant spores will not be detected; lack of specificity	[22, 140]
Bioorthogonal noncanonical amino acid tagging (BONCAT) with click chemistry	Measuring translational activity via synthetic amino acid incorporation into proteins	Y	Many (e.g., FISH, AFH, flow cytometry, FACS, MDA, 16S rRNA gene sequencing, presumably, other DNA amplification and sequencing techniques and protein-based techniques)	Presumably many; thus far, deep-sea methane seep sediments	Unknown	Presumably many; thus far, some archaea and Gram − bacteria, including slow growing	Can reveal actively translating microbes in consortia and, in combination with downstream approaches, their phylogeny; insights into micron-scale interactions	Application to microbial ecology is relatively new; broad applicability is presumed but not yet proven	[176, 175]
Isothermal microcalorimetry (IMC)	Measuring heat flow	Y	Many (the method is nondestructive)	Many, including lakes, marine sediments, and soils	Y	Many (any actively metabolizing organisms generating heat)	Will measure any sufficient metabolic activity	Can only be applied to slow processes because of assay ramp-up time; possible false positive signatures (e.g. degradation of media)	[183]
Stable-isotope probing (SIP)	Tracing isotopically labeled substrates through an active microbial community	Y	PCR, FACS	Many	N	Many (e.g., archaea, Gram + bacteria, Gram − bacteria, fungi, spores if actively incorporating substrates, and replicating viruses)	Can determine metabolic activity and phylogeny in the same sample; can help to identify community members involved in the metabolism of specific labeled compounds of interest	Long incubation times may be necessary; labeled substrates can be expensive; relatively large amount of biomass needed; the label can move through trophic networks during the incubation, so careful interpretations are necessary	[153]
Proteomics/metaproteomics	Identifying proteins via mass spectrometry	N	N/A, unless initial sample is split for multiple purposes	Any, given sufficient protein yield and quality	N	Many (e.g., archaea, Gram + bacteria, Gram − bacteria, fungi, replicating viruses; can also measure viral structural proteins, which do not necessarily indicate infectivity)	Can identify actively expressed proteins and metabolic pathways	Requires exact protein sequence to be present in database for identification; often lower throughput than nucleic acid sequencing approaches	[161]

“Many” means that most of the possibilities for this category have been shown to be, or are likely to be, compatible; where practical, we have added examples from the literature. For abbreviations, see the list of abbreviations at the end of the main text

^a^We did not find evidence for attempts of this application for this technique

## Common techniques for viability assessments

### Culture-based techniques

Successful culturing is a clear indication that an organism is alive, but unsuccessful culturing is not proof of the lack of life. For example, microorganisms may fall under a category, first described by Huai-Shu and colleagues [25], of “viable but non-culturable” (VBNC), meaning that they are alive but do not divide using common culturing techniques [26, 27]. A VBNC condition has been observed for organisms that can no longer form colonies under the test conditions, such as those inhabiting spacecraft clean rooms [28], or damaged cells that are no longer able to divide but are still alive [29]. Similarly, slow-growing and/or quiescent cells may be difficult or impractical to culture [30, 31].

While the statistic that “99% of microbes are uncultivable” has been popularized, the reality is typically more complex, as this is more of a comment on human technology than a condition of the microbes [32]. Still, it is true that many microbes are difficult to culture, either because of innate fastidiousness or because of the time that it would take to determine acceptable culturing conditions [33, 34]. Therefore, in many cases, the impetus, facility, and/or time necessary for developing an appropriate cultivation technique may not be available. In addition, waiting for the detection of live cells through culturing imposes a time delay, thus providing a practical incentive for the development and use of more rapid methods, particularly when health and safety are at risk. For these reasons, culture-independent methods have been developed for use in conjunction with, or even supplanting, culture-dependent methods [21, 35–37]. For example, culture-dependent and culture-independent assays for quantifying viable microbes have been reviewed in the context of probiotics, with a focus on identifying and optimizing assays for enumerating microbes across metabolic states, including VBNC [38].

### Techniques based on membrane integrity

**Fig. 2:**
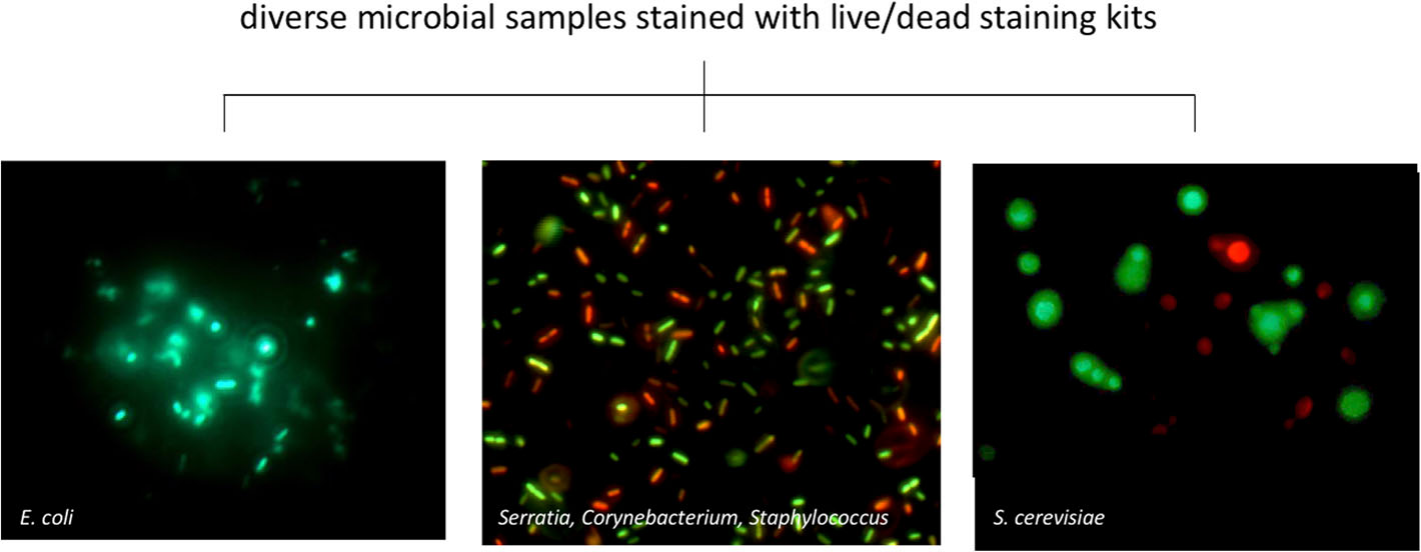
Example of Live/Dead staining kits applied to two bacterial samples and a eukaryotic sample. (A) A pure culture of *E. coli* was grown in LB medium overnight at 37 °C to an OD_660_ of 0.4. The cells were incubated with 100 mM H_2_O_2_ for 1 h at 37 °C. The sample was then stained with the LIVE/DEAD BacLight Bacterial Kit-L-7007 (Invitrogen, Grand Island, NY, USA) for microscopy according to the manufacturer’s instructions. A 10-μL aliquot was examined by fluorescence microscopy on a Carl Zeiss Axioskop using a filter with an excitation 488 nm and emission 528 nm. The live cells fluoresce green. (B) A developing biofilm on a glass slide created by incubating the slide in a solution containing three bacterial species: (1) *Serratia marcescens* ATCC 14756, (2) *Corynebacterium xerosis* ATCC 373, and (3) *Staphylococcus epidermis* ATCC 14990. It was stained using the LIVE/DEAD BacLight bacterial viability kit (PI/SYTO) [Molecular Probes]. Here, the live cells fluoresce green while the dead cells fluoresce red. (C) Yeast cells were stained with the LIVE/DEAD Yeast Viability Kit L-7009 (FUN 1 cell stain). The yeast were grown overnight in Sabouraud medium at 28 °C and then incubated with 100 μM H_2_O_2_ for 1 h. The samples were stained with FUN 1 cell stain according to the manufacturer’s instructions. The cells (10 μl aliquots) were viewed under a fluorescence microscope Axioskop (Carl Zeiss) with an excitation 489 nm and emission 539 nm. In contrast to the images of bacteria, here, the live cells form red fluorescent structures, while the dead cells are distinguished by a diffuse, green fluorescence. *E. coli* and *S. cerevisiae* micrographs were obtained by coauthor Balk, and the mixed bacterial micrograph was obtained by coauthors Adams and Lymperopoulou

The outer cell membrane is critical to all life on Earth, as it defines the individual cell, provides cellular compartmentalization, and is the physical, chemical, and biological interface with the outside world. Thus, membrane integrity is considered to be a biomarker for viable cells because cells with compromised membranes are—or will soon be—dead. Fortunately, cell membrane integrity can be measured in many cases, for example, via selective stains coupled with microscopy (Fig. 2) or, more recently, coupled with sequencing approaches [39]. Using membrane integrity-based fluorescence staining coupled to flow cytometry, the proportion of living microorganisms was reported to be 70–80% in drinking water [40–42] and 50–60% in the surface waters of freshwater, estuarine, and coastal marine stations [43, 44]. However, there are at least two limitations to this approach. First, techniques based on membrane integrity may result in an overestimation of a “snapshot” of viable cells because lethal stress may not lead to immediate cell membrane disintegration. Second, the dyes that are typically employed to assess membrane integrity may be ineffective against cells with a hardy membrane or cell wall, such as spores [40, 45, 46].

#### Propidium iodide (PI) viability stain

**Fig. 3:**
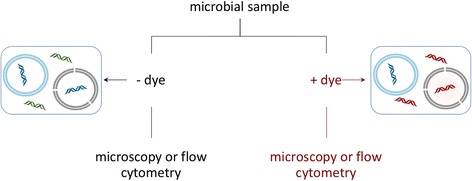
Live/dead staining workflow, propidium iodide (PI) example. In this technique, the sample is divided in two. One sample (*left side*) is stained with a total nucleic acid stain and used for cell enumeration, in which the live (*blue* membrane) and dead (*black* membrane) cells cannot be distinguished from each other, resulting in a stain of all nucleic acids. In the propidium iodide (PI) stained sample, the stain permeates compromised cell membranes, staining both cells presumed to be dead or in the process of dying (*black* membrane) and extracellular DNA or DNA, with PI-stained DNA colored red. Live cells with intact membranes (*blue* membrane) are not stained. In both types of samples, localization of stains within cells allows for enumeration, with stained free DNA relegated to background fluorescence. A comparison of counts from stained and unstained samples can be used to estimate the number of living cells. Alternatively, a single sample can be prepared with both a total nucleic acid stain and propidium iodide for counts of living and dead cells in the same preparation (not shown)

One of the most commonly used fluorescent stains to determine viability by membrane integrity is propidium iodide (PI, Table 1), although other stains that work in a similar matter, such as SYTOX Red Dead (ThermoFisher, Grand Island, NY, USA), are marketed for flow cytometry. PI is a red-fluorescent dye (excitation/emission maxima 493/636 nm, respectively) that usually does not permeate cells with intact membranes. If the cell membrane is compromised, PI usually crosses the cell membrane and then binds to the internal nucleic acids (Fig. 3) [47]. Because of its fluorescent properties, PI can be used to detect membrane-compromised cells via epifluorescence microscopy (Fig. 2), flow cytometry [48, 49], and fluorometry [50]. For example, the LIVE/DEAD BacLight Bacterial Viability Kits (ThermoFisher, cat # L-7007) use PI to stain membrane-compromised cells in combination with SYTO 9 (a green fluorescent total nucleic acid stain) to stain all cells (Fig. 2, *Escherichia coli* image). Similar kits are available for eukaryotic cells, such as yeast (Fig. 2, yeast image). For example, the FUN® 1 kit (ThermoFisher, cat # L-7030) is a two-color fluorescent viability probe for yeast and other fungi, and the LIVE/DEAD Reduced Biohazard Cell Viability Kit #1 and the ReadyProbes® Cell Viability Imaging Kit (Blue/Red) (both available from ThermoFisher) target eukaryotic cells, including protists.

PI has been used for a range of applications, including for lab-based tests and field samples, and on a variety of sample and organism types, such as marine bacterioplankton [47] and soil bacteria [51]. Kits for distinguishing between living and dead cells in microbial communities become even more useful in combination with quantitative techniques, as described below. The combined techniques have been applied to a number of bacterial species, most of which are known pathogens [52, 53].

PI methods have some limitations and are therefore not necessarily the best staining choice. For example, in some cases, PI can stain both growing and non-viable cells, as demonstrated for *Sphingomonas sp*. and *Mycobacterium frederiksbergense* [54]. In addition, PI has not been shown to be compatible with subsequent DNA sequencing.

#### Viability PCR via ethidium monoazide (EMA) and propidium monoazide (PMA)

The DNA amplification-based solution for the preferential detection of live cells is called “viability PCR.” Although this technique is likely to be broadly applicable to microbial studies, it is as yet seldom mentioned in the microbial ecology literature. As such, we provide more methodological details here than for other techniques that are more widespread in the literature.

**Fig. 4:**
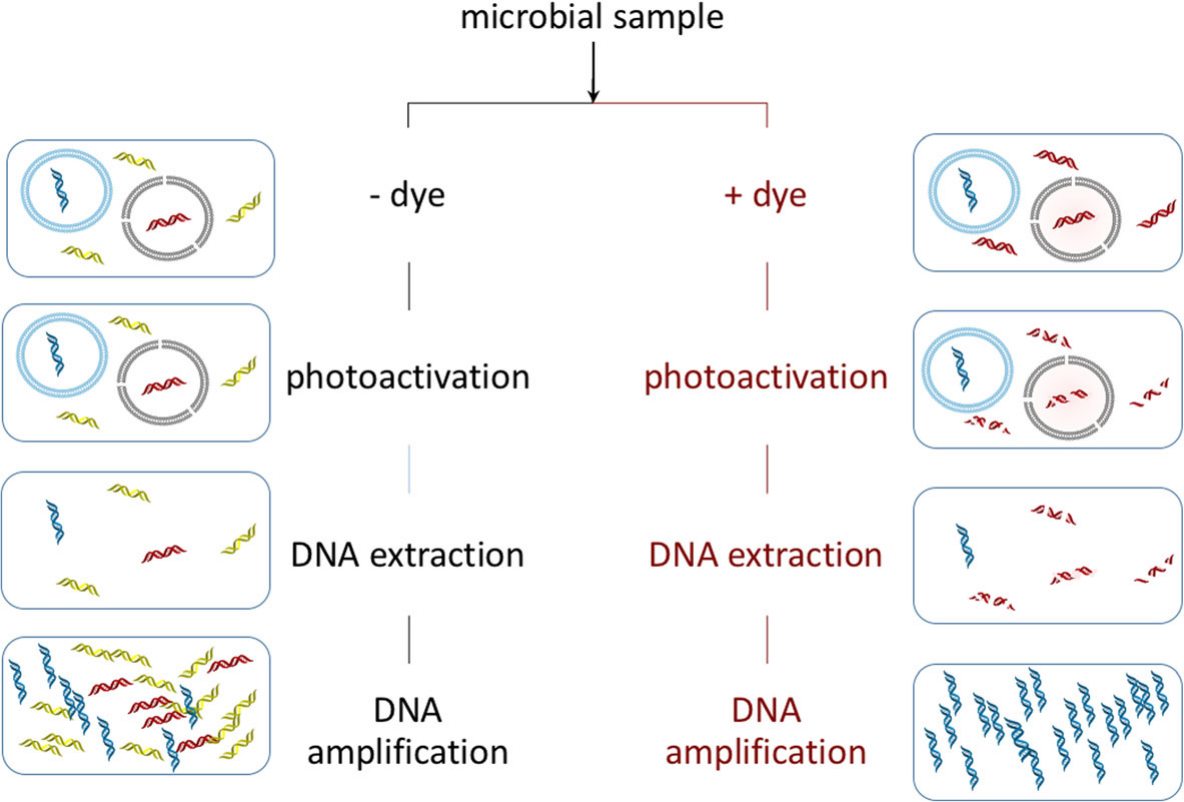
Viability PCR workflow (e.g., using EMA, PMA, or similar dyes). The initial sample is divided in two. One sample (*left side*) remains untreated, leaving total DNA—including extracellular DNA (*yellow*) and DNA in living (*blue* DNA, *blue* membrane) and dead (*red* DNA, *black* membrane) cells—relatively intact and available for downstream applications. The other sample (*right side*) is stained with a viability dye that binds to free DNA and to DNA in cells with compromised membranes. Upon photoactivation in the treated sample, bound DNA is degraded, such that it is no longer a suitable template for amplification. After amplification, a comparison of treated versus untreated samples can reveal relative proportions and/or types of living and dead microorganisms (e.g., via qPCR and/or DNA sequencing, respectively)

In viability PCR, shown diagrammatically in Fig. 4, samples are first treated with a viability dye, such as ethidium monoazide (EMA) or propidium monoazide (PMA), which, as with PI, penetrates damaged cell membranes (Table 1). Once inside the cells, these nucleic acid intercalating dyes bind to DNA. Upon exposure to bright light (described below), the dyes form covalent bonds [55], and the photoactivation results in irreversible damage to the nucleic acids, including strand breaks [56]. When the DNA from a treated sample is amplified, only DNA from cells with intact membranes should be amplified, as the degraded DNA from extracellular DNA and from cells with compromised cell membranes should provide poor templates for DNA amplification [57–59]. EMA has been used to differentiate live from dead microorganisms via flow cytometry, and, subsequently, in viability PCR, allowing for the first selective detection of nucleic acids from intact cells [60].

One drawback for EMA is that it has different effects on different species. Intact cells from both some Gram-positive and Gram-negative species have been known to take up the dye, including *E. coli* O157:H7, *Streptococcus sobrinus*, *Micrococcus luteus*, *Staphylococcus aureus*, and *Mycobacterium avium* [57]. As a result, viable cells appear to be dead and are counted as false negatives. Additionally, EMA can show a higher cytotoxicity than PMA in some species, meaning that the dying process itself may kill the very cells it should distinguish as living. For example, in *Listeria monocytogenes* and *Legionella pneumophila*, higher cytotoxicity was observed when using EMA, relative to PMA [58, 59]. This observation has been attributed to the fact that EMA has only one positive charge and therefore more easily penetrates bacterial membranes than PMA, which has a double positive charge [61]. However, this may also result in less efficient suppression of dead cell signals by PMA relative to EMA, because PMA may not as easily permeate cells with only slightly compromised membranes. Although this does not apply to every bacterial species tested, fourfold more PMA than EMA was necessary to achieve the same degree of signal reduction for dead *Legionella* cells [62]. Some physiological states of live cells also support changes in membrane permeability, influencing the uptake of EMA by rapidly dividing and senescent cells [63]. Although this has not been tested for PMA, this likely applies to PMA as well. Intact cell concentrations obtained with PMA-quantitative PCR (PMA-qPCR) can probably be interpreted as maximal values because the abundance of killed cells can be underestimated, as reported for heat-killed *Listeria innocua* in comparison with plate counts and fluorescence microscopy [64]. Overall, PMA is more selective than EMA in its exclusion of damaged cells, so we will focus the rest of this section on PMA.

The PMA reagent is available commercially from several vendors, including GenIUL (PhAST Blue), Qiagen (BLU-V PMA Viability Kit), and Biotium (PMA-Lite). In addition, a new variant on PMA, PMAxx (Biotium, Inc., Hayward, CA, USA), has recently become available, though the chemical composition of PMAxx and its relationship to PMA are proprietary. Testing of PMAxx has been limited, but preliminary results suggest that the same procedures can be used as for PMA, and it may perform better than PMA in at least some cases [65].

To assess the relative proportions of living and dead cells in a community by DNA proxy, samples are handled in duplicate, one treated with PMA and one without PMA, followed by photoactivation, as shown in Fig. 4. The dye excitation maximum is at 464 nm, allowing for the use of blue light-emitting diodes (LEDs) with an emission at 465 nm. After PMA treatment, DNA is extracted, and amplification is typically performed by PCR. PCR-based procedures found to be compatible with PMA treatment include qPCR [66, 67], microarrays [20, 68], denaturing gradient gel electrophoresis (DGGE) [69], and amplicon sequencing [70–72]. In addition to PCR, successful PMA treatment has been reported in combination with loop-mediated isothermal amplification (LAMP) [73], multiple displacement amplification (MDA) [74], and metagenomic library construction for low-biomass clean room samples [39].

Although further methods development will be required to assess the applicability of PMA across organism and sample types, encouragingly, a variety of organisms and environments have already undergone successful PMA treatment. For example, PMA treatment of methanogenic archaea has recently been demonstrated in both pure cultures and in some sediment and soil samples [75], and PMA results from complex microbial communities in a variety of soil types have been reported [15]. In a study involving PMA treatment followed by amplicon sequencing of bacterial communities in sputum samples of cystic fibrosis patients, treating samples with PMA resulted in the detection of a higher diversity of species and better representation of rare community members, relative to untreated samples [76].

The successful PMA treatment of low-biomass samples has also been demonstrated [20, 39, 72]. An additional application of PMA that is particularly useful for low-biomass samples is in the removal of contaminating extracellular DNA from commercial PCR reagents [77], to which low-biomass samples may be particularly sensitive. Following reagent treatment with EMA [78] or PMA [79], contaminating DNA was no longer amplified, and the combination of PMA treatment of both PCR reagents and microbiological samples was shown to increase the probability of detecting low numbers of live cells [79].

PMA treatment is relatively easy to perform but lacks a standardized procedure. Light exposure systems include commercially available LED photolysis devices, such as the PMA-Lite LED Photolysis Device (Biotium, Inc., Hayward, CA, USA), PhAST Blue (GenIUL, Terrassa, Spain), and the BLU-V System (Qiagen, Valencia, CA). However, these specialized LED systems are currently relatively limited in terms of throughput and format (e.g., treatment can only occur in microcentrifuge tubes). The optimal wavelength for the PMA assay is 464 nm, and successful use of commercially available halogen lamps has been reported [15]. However, these halogen lamps can have variable light intensities and unknown spectral properties, which may make results difficult to interpret and reproduce if emission wavelengths are not measured. Also, heat from halogen lamps might compromise membrane integrity and dye permeability and/or could melt plastic tubes, so use of halogen lamps may require samples to be incubated on ice or in a cooling incubator and/or to be incubated in stages with the light cycled on and off, as in [15].

In addition to the light spectrum and intensity, factors that can influence the effectiveness of PMA assays include the source and concentration of dye, microbial community composition, dye incubation time and temperature, sample turbidity, and the clay and salt contents of the sample [61, 75, 80]. As light penetration is critical to the success of PMA treatment, the turbidity of the sample must be in a range that allows for efficient light penetration during the photoactivation step. This is of particular concern for particulate-laden samples, such as soils and sediments. Different approaches to improve the efficiency of PMA-PCR have been developed, including:

A) *Amplification of longer sequences*: Increasing the amplicon length increases the probability that at least one dye-binding event will have occurred in a given stretch of DNA from dead cells, thereby increasing signal suppression from those DNA sequences. This has been demonstrated for both EMA [81–84] and PMA [79, 83, 85].
B) *Performance of dye incubation at elevated temperature*: Dye permeability of lipid bilayers depends to a large extent on temperature, with elevated temperatures during sample treatment increasing dye uptake. Suppression of signals from damaged *Salmonella typhimurium* and *L. monocytogenes* were improved by performing PMA treatment at temperatures up to 40 °C, while signals from live cells were not affected [80]. It was suggested that dye incubations at temperatures exceeding the ambient temperatures of organisms by 10 °C might be a reasonable strategy to increase PMA-qPCR efficiency.
C) *Extending dye incubation time and increasing dye concentration*: Treatment of a sample with higher concentrations of dye might be necessary when the sample has substances that exhibit a dye demand. Increasing dye concentrations and extending dye incubation times have been shown to increase treatment efficiency [80].
D) *Incubation in the presence of facilitating substances*: Co-incubation of cells with PMA and the bile salt, deoxycholate, has been shown to improve PMA treatment efficiency for Gram-negative bacteria, but deoxycholate is not compatible with bile-sensitive Gram-positive bacteria [80, 86]. Also, dimethyl sulfoxide (DMSO) and ethylenediamine tetraacetic acid (EDTA) are known to affect dye permeability through membranes [87, 88]. Although signal suppression of damaged cells can be achieved by increasing the concentrations of these substances, it is important to ensure that there is no detrimental effect on live cell signals or downstream processing steps.
E) *Multiple dye treatments*: Repeated sample treatment with a viability dye (i.e., the addition of dye, followed by photoactivation, then additional rounds of dye addition and photoactivation) has been shown to improve signal suppression for heat-killed *M. avium*, which, due to its thick cell wall and the presence of mycolic acids that affect PMA penetration, is less amenable for dye uptake through compromised membranes than other bacterial species [89].

#### Detection of stained cells: epifluorescence microscopy versus flow cytometry

The reliability of the methods for detecting live versus dead cells depends not only on cell physiology but also on the limits of the instrumentation used to analyze the cells. Many of the stain-based techniques can be used with either microscopy or flow cytometry. For microscopy, the sample is stained and applied to a microscope slide, and the user performs counts manually or with the assistance of image-processing software [90, 91]. For flow cytometry, the user loads the sample into the instrument in an aqueous solution, and biological particles are automatically counted by the instrument, with some opportunities for manually constraining the size, shape, and/or fluorescence intensity of the counted particles [92]. However, these user-constrained settings for identifying signals from microbial cells are often based on the behavior of a standard, such as a pure culture, and the broad applicability of the staining properties of the standard, including any growth media, to complex communities is likely to be unknown. This means that there could be false positives in the standard itself, which could skew counts in the actual sample, and/or that both false positives and false negatives could appear in the sample because of its distinct chemical and/or biological (e.g., cell size) properties, relative to the standard. Some of these issues are unavoidable limitations of the approach, but controls, including no-sample blanks, media-only samples, and a dilution series of standards (in media serially diluted with pure water), may help to disentangle false positives in the standards. Multiple standards across a range of cell sizes could also be considered to increase the likelihood of encountering similar cell types in the sample as in the standards.

When analyzing environmental samples, epifluorescence microscopy and flow cytometry share many of the same challenges. For example, certain dyes, including both total nucleic acid stains and live/dead stains, such as PI, readily adhere to particles and substrate material, resulting in increased non-specific binding and background fluorescence and, therefore, false positives (which can also result from simple scattered light, irrespective of the dye) [92, 93]. In addition, dyes often used for viability staining require significant optimization to ensure compatibility with a given sample type or microbial population, suggesting that a single dye is unlikely to work for all of the cells in a complex microbial community [21]. One advantage of epifluorescence microscopy over flow cytometry is that the user may be capable of visually distinguishing cells from non-biological material, and epifluorescence microscopy can be useful as a diagnostic tool to visually inspect how a dye interacts with a given sample when developing protocols for flow cytometry. In addition, as long as counts are sufficiently above observations on blank controls (which should be included for any reported counts from flow cytometry or epifluorescence microscopy), epifluorescence microscopy can be used to analyze low-biomass samples (e.g., air samples [94]) that might be more difficult to analyze with flow cytometry, in which low numbers of “events” (microbes passing in front of the light source) may be difficult to distinguish from background. The greatest disadvantage of epifluorescence microscopy, relative to flow cytometry, is the low throughput; each sample takes time to prepare and analyze, including the time needed to count ~10 or more fields per sample [91]. Although image-processing software is available for quantitative microscopy, it works by evaluating intensity levels per pixel, such that autofluorescence and non-specific dye binding in environmental samples may make it challenging for such programs to distinguish cells from background.

Conversely, the greatest advantage of flow cytometry is the throughput (thousands of cells can be counted per second, and tens of samples can be prepared and counted in a day). However, in order to be confident in measurements from flow cytometry for environmental samples, particularly from complex matrices like dust, soil, and sediments, significant methods optimization may be needed at the start of an experiment. In addition, these types of samples may be difficult to homogenize for passage through the narrow aperture required for flow cytometry. Contamination from the instrument (e.g., from previous samples) is also a concern. Therefore, rather than solely reporting final counts, documentation of methods optimization, including visual evidence of how standards and blank controls compare to samples, is strongly encouraged for flow cytometry applied to environmental samples, both for community enumeration and for live/dead counts.

### Techniques based on transcription (RNA analyses)

**Fig. 5:**
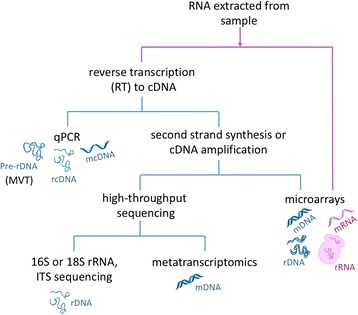
Summary of RNA-based techniques. Techniques that use RNA directly have *pink pathway lines*, and those using complementary DNA (cDNA, after retrotranscription) and double-stranded DNA (DNA, after second-strand synthesis or amplification) are *colored blue. MVT* is molecular viability testing

There is an increasing use of ribonucleic acids (RNA) to target the active members of microbial communities. The use of RNA as a molecular target for living microbes makes biological sense, since transcription is among the first levels of cellular response to stimuli (and, in the case of some viruses, transcription can represent active viral replication), and RNA has a much shorter average half-life than DNA (see below), such that RNA collected from an environmental sample most likely represents living microbes. RNA-based methods (Table 1) have been used in a variety of environments, across a broad spectrum of microorganisms, and via a number of techniques, including microarrays, qPCR, 16S ribosomal RNA (rRNA) sequencing, and (meta)transcriptomics, each typically requiring an initial reverse transcription step (summarized in Fig. 5) [95–102]. Each of these approaches can potentially reveal the identity of viable microorganisms. As messenger ribonucleic acid (mRNA) is extremely short-lived (with an average half-life of minutes in active cells and even less as a free molecule in the environment [103, 104]), approaches focused on mRNA can track specific microbial metabolic responses on short timescales [95, 102, 105]. Compared to mRNA, rRNA has a half-life of days [106] and is more abundant in cells (approximately 18% of the dry weight of a bacterial cell [107] and up to 90% of total cellular RNA is rRNA [108]). In addition, rRNA may allow for a more accurate taxonomic identification of populations. Thus, rRNA approaches may be more successful than mRNA approaches, particularly for low-biomass samples.

There are both theoretical and practical concerns with RNA-based methods. Interpretation of 16S rRNA sequencing analyses is complicated by the fact that there is not an absolute correlation between the concentration of rRNA and cell activity or growth rate [105]. The relationship between rRNA and cell state can vary both within and between populations, for example, due to life strategy (e.g., dormant or persistent cells). Dormancy is a phylogenetically widespread strategy for surviving stressful conditions that requires a substantial energetic investment [109]. In fact, in the manufactured environment and other low-biomass systems, the number of dormant cells is likely to be high, due to low resource availability [109, 110]. Cells entering dormancy contain high numbers of ribosomes, which they may keep in preparation for emerging from a dormant state [105]. Confounding this, the concentration of ribosomes can depend on how the state of dormancy originated [95]. In these cases, although the rRNA concentration does not directly correspond to current activity, it indicates that a given organism is not only viable but also potentially capable of a rapid response in a new environment. Therefore, in terms of rRNA relative abundances, tracking longitudinal changes within and across taxa can yield important insights into community dynamics [95].

From a practical perspective, processing RNA is more complicated than processing DNA. The highly labile nature of mRNA can make sample processing a challenge, and losses of up to 80% of the total mRNA have been reported during sample preparation [111]. Such extreme sample loss is particularly important to consider for low-biomass studies, which may have insufficient RNA yields [20]. Some of the technical challenges that arise with processing RNA include uncertainties with regard to how sample preparation, extraction methods, RNA preservation, and retrotranscription affect the resulting RNA signal. To account for the sample loss, the RNA or rRNA concentration is typically normalized to the appropriate DNA concentration to calculate either the overall RNA:DNA ratio or the more specific rRNA:rRNA gene ratio [105], and the mRNA is normalized by the mass of the total RNA analyzed [111]. This normalization approach is only valid if the degradation rate is uniform over the population of RNAs, but such degradation rates are often unknown, particularly for mRNA. Some studies of eukaryotic cells have indicated that the GC content of the RNA is an important factor in influencing the degradation rate [112–114].

In order to minimize any potential degradation bias, alternative approaches, such as the molecular viability test (MVT), are being developed. MVT targets precursor rRNA (pre-rRNA) [115, 116], which shows a turnover rate similar to mRNA but is a more stable molecule. In growing bacteria, pre-rRNA can account for more than 20% of the total RNA [116]. However, MVT requires knowledge of the target region sequence and is therefore not yet conducive to most microbial community ecological studies.

## Using molecular approaches to determine total or live/dead abundances

As a prerequisite for accurate live/dead enumeration, accurate enumeration of untreated microbial communities is necessary. In addition to the aforementioned epifluorescence microscopy and flow cytometry approaches, which can be used in combination with fluorescent dyes to count cells, molecular methods, such as qPCR and RT-PCR, can measure nucleic acid abundances as proxies for cellular abundances. In viability qPCR, PMA (or EMA)-treated samples are quantified and compared to the same non-treated samples. Here, we review basic qPCR enumeration approaches for microbial communities, including considerations that are applicable both to untreated samples and to the separate quantification of living and/or dead cells (e.g., following PMA treatment). We also introduce digital qPCR as a high-throughput method that is likely to be useful for future live/dead abundance measurements.

### Quantitative PCR (qPCR)

Quantitative PCR is a means of measuring the DNA concentration, or number of copies of a specific gene or genetic region in a sample, relative to a known set of standard DNA concentrations (or calibrators, in the case of relative quantification), and this is accomplished through real-time assessments of the amount of DNA replicated during a PCR reaction. RNA amplification can be similarly quantified through RT-PCR, and, in the interest of brevity, we focus on the approach for DNA. DNA replication is measured as the incorporation of a fluorescent nucleic acid stain, for example, a double-stranded DNA-intercalating dye like SYBR Green or a fluorescently labeled probe, during the PCR reaction. Because the cycle from which fluorescence is detectable (the threshold cycle, Ct, or quantification cycle, Cq) is related to the amount of DNA in the sample, the Ct in PMA-treated and non-treated samples can be used to quantify the living microbial community (PMA-treated), the total microbial community (PMA-untreated), and, to some degree, the dead microbial community (the difference between PMA-untreated and PMA-treated).

The advantages and disadvantages of different qPCR techniques have been extensively reviewed elsewhere (e.g., [117]), and we highlight the fact that probe-based qPCR is likely to be problematic for complex community studies because probe-based techniques rely on sequence conservation. Therefore, non-specific DNA-intercalating dyes are likely to be a more appropriate choice for qPCR of microbial communities. Also, the efficiency of DNA extraction, the presence of PCR inhibitors, different gene copy numbers, and, if applicable, the efficiency of cell purification prior to DNA extraction have all been highlighted as important considerations for the interpretation of qPCR results from environmental samples [118, 119].

Quantitative PCR techniques for low-biomass samples have been benchmarked [118] and applied in a number of studies of air and the built environment for measuring bacterial and fungal concentrations [119–122]. Although the universal 16S rRNA gene primers used for detecting bacteria in some of these studies are also known to amplify at least some archaea, these primers have not been specifically benchmarked for the enumeration of archaea. Quantitative PCR has been used successfully in combination with PMA for estimating live/dead concentrations of microbial cells in low-biomass samples [67], and with some methods development, this combination of techniques is likely to be broadly applicable across environments.

### Digital PCR

Application of digital PCR technology is very similar to traditional qPCR and has been implemented to quantify biomass in a variety of microbial systems [123–126]. Digital PCR is based on the partitioning of template molecules into many replicates, creating a limiting dilution [127, 128]. Replicates contain approximately one or no templates per reaction. Partitioned replicates then undergo thermocycling to end point, and the concentration of starting material is determined via a Poisson statistical analysis, which uses the counts of positive and negative replicate reactions. The more replicates, the higher the accuracy and confidence of the counting estimate [129]. Currently, there are three commercially available methods for creating replicates based on microchip [130], emulsion and bead [131], and oil and water separation techniques [129, 132].

Several studies have benchmarked digital PCR results against established qPCR assays and consistently show digital PCR to be more accurate with lower variability across replicates [123–125, 133–135]. While the enhanced performance of digital PCR is certainly a boon, perhaps the most exciting feature of the technology is that there is no need to run internal standards, making results comparable across studies and laboratories. Recent advances in digital PCR technology have reduced the cost per reaction while increasing sensitivity and accuracy, making it an attractive option for counting microbes. Though live/dead assessments using digital PCR have not been reported, they should be essentially the same as combinations of live/dead assessments with traditional qPCR.

## Viability assays based on cellular metabolism and other properties

In principle, cellular metabolism should be an ideal assay for viable organisms, as organisms that are not metabolically active are either dead or in a dormant (e.g., spore) state. This was recognized by the creators of NASA’s biological experiments aboard the Viking Landers in 1977, the first life detection attempt on Mars [136, 137]. On Earth, a number of other methods utilize dyes to detect different aspects of active cellular metabolism, which may be used to infer and/or assess the viability of microorganisms. While it is beyond the scope of this review to cover all of the many dyes and stains used for live/dead assessments, these dyes (many of which are compatible with epifluorescence microscopy and/or flow cytometry) have been reviewed elsewhere [22, 24].

### ATP as a biomarker for viable microorganisms

ATP, used as energy currency for metabolic activities by all living organisms, can also be used as an indicator of viability and cellular activity (Table 1) [138–140]. Many commercially available ATP detection kits have been used for cell viability and cytotoxicity measurements for decades, including in the food industry, for drug discovery, in assessments of drinking water, and in soil [139, 141–144]. The method typically involves the addition of an ATP-releasing reagent to lyse cells and release ATP, which, in the presence of luciferase, reacts with the substrate, d-luciferin, to produce light. The light intensity is then measured as relative light units (RLU), which is interpreted as a measure of ATP concentration [145–147]. Though a direct correlation between the total number of cells and RLU cannot be readily established, this provides a reasonable estimate of cellular activity. For example, the measured concentrations of bacterial ATP in different aquatic microbial communities correlate well with the concentrations of living cells when the ATP measurements are complemented by other tools (e.g., live/dead staining via PI in combination with SYTO9 and flow cytometry or microscopy) [22].

One drawback of using ATP assays as a proxy for living cells is that they may overestimate the total ATP counts because they also measure exogenous ATP. One commercially available ATP assay kit, CheckLite (Kikkoman, Japan), removes the exogenous ATP first enzymatically, followed by cell lysis and an assay of intracellular ATP. This selective detection of intracellular ATP was used to measure the cleanliness of low-biomass clean room environments [140], and it may be broadly applicable across other sample types. Recently, ATP measurements were used in indoor environments to show that doorknobs with visible debris tended to have more ATP (interpreted as higher metabolic activity) than “clean” doorknobs without visible debris [148].

### Isotope probing

**Fig. 6:**
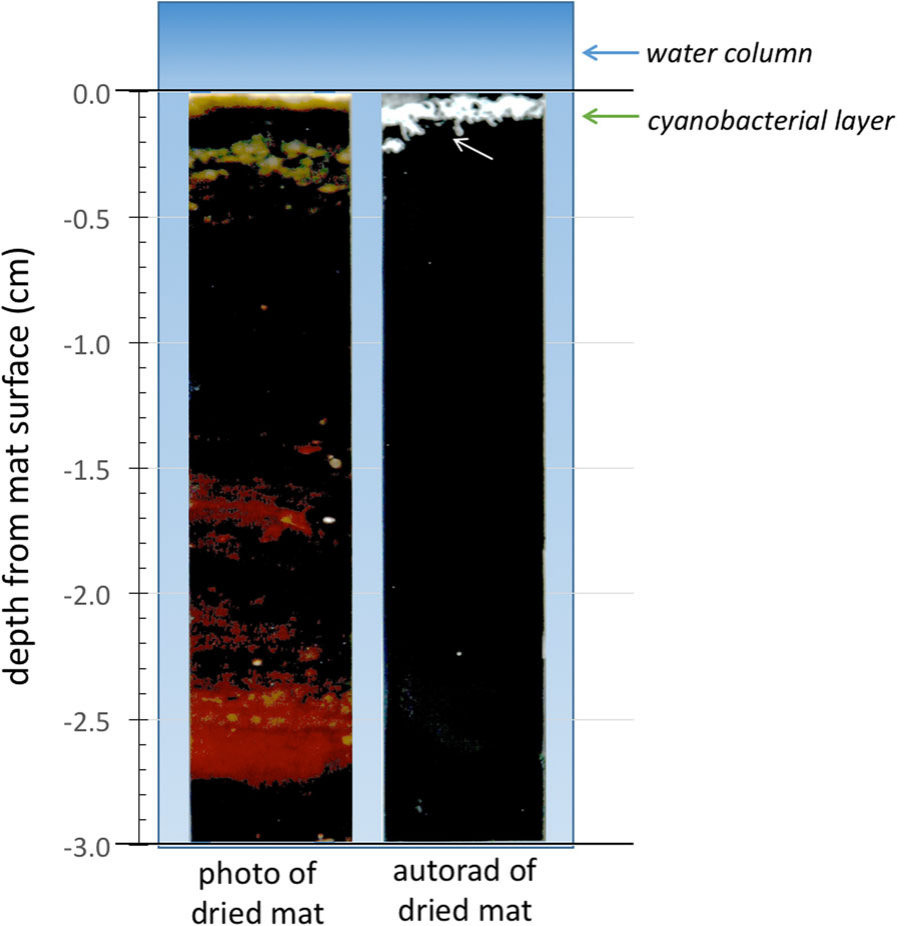
Autoradiography. The incorporation of radiolabelled isotopes by actively metabolizing organisms subsequently detected at the community level with scintillation counting, or at the individual level with microautoradiography, allows the precise identification of not only actively metabolizing members of an ecosystem, but metabolic type. Here, Rothschild and Mancinelli [201] sought to identify the location of the actively photosynthesizing members of a laminated microbial mat sample without destroying the fabric of the mat. Whirlpak® bags containing mat samples and water supplemented with radiolabelled 1 μCi/ml NaH^14^CO_3_ (New England Nuclear NEC 086H) were sealed and returned to the collection pond to incubate under in situ temperature and light levels, and then formalin was added to kill cells. In the lab, the samples were washed in acidified water, sliced to a thickness of ~2 mm with a gel slicer, and then frozen between two glass plates, which were removed prior to autoradiography. The frozen mats were exposed to X-ray film for 2–14 weeks at −80 °C. The developed film was placed in a photographic enlarger and used as a negative to print the image on the right and stands in contrast to the photograph of the frozen mat on the *left*. The *white areas* in the autoradiography panel correspond to acid-stable ^14^C incorporated into the mat sample, indicating the actively photosynthesizing community members

The active incorporation of radioactive metabolites has been used to detect metabolizing cells via microautoradiography for decades. Tritiated thymidine has been widely used to measure DNA synthesis, and radiolabeled (usually ^14^C) leucine has been used for protein synthesis [149]. One concern is that some cells will take up thymidine from the medium but, rather than incorporating it into DNA, will metabolize it. Thus, if total radioactivity is used as a proxy for DNA synthesis (secondary production), it may be misleading. Further, there are many cells that will not take up thymidine from the medium, and the rates of uptake and incorporation may vary among species. This led to a technique suitable for field studies based on the incorporation of ^33^PO_3_ into DNA [150]. For photosynthetic organisms, acid-stable incorporation of ^14^C-labeled bicarbonate has been used for bulk analysis (Fig. 6), while ^14^C-labeled acetate (or other sugar) can be used for heterotrophs. Similarly, ^14^C leucine incorporation into proteins has been used to measure translational activity in both prokaryotes and eukaryotes [151]. Microautoradiography has been used in conjunction with fluorescence in situ hybridization (FISH) [152].

While radiolabeling is highly sensitive, safety concerns and specialized equipment needs have turned most researchers away from these techniques. Stable-isotope probing (SIP, Table 1) now allows for a safer means of performing similar analyses. Community stable-isotope probing (SIP) methods can allow for the identification of active taxa and metabolisms by tracing the incorporation of labeled isotopes (e.g., ^13^C, ^2^H, ^15^N) into a microbial community. Following the incubation of an environmental sample with a labeled substrate, any labeled biomolecule can theoretically be targeted for detection. Nucleic acid-based SIP is perhaps most commonly employed, given the phylogenetic resolution provided by downstream sequencing approaches. In DNA- or RNA-SIP techniques, labeled and unlabeled fractions can be separated by density-gradient centrifugation, and the populations in the resultant bands identified by 16S rRNA gene profiling [153, 154] or metagenomics [155]. While powerful in its ability to distinguish metabolic activity and phylogeny simultaneously, nucleic acid-based SIP techniques require a relatively large amount of biomass, labeled substrate (which is often expensive), and a labeling step (i.e., an incubation).

Nano-scale secondary ion mass spectrometry (NanoSIMS) has also been used to detect the uptake of labeled substrates as an indicator of metabolic activity, allowing for single-cell resolution, which can be useful for describing rare members of the community [156, 157]. In combination, NanoSIMS and FISH can be used to link metabolism to phylogeny, allowing for the identification of active members of a microbial community [158, 159]. However, in addition to the aforementioned limitations of SIP, NanoSIMS is low-throughput, the instrumentation to perform the analysis is often not readily accessible, and coupling phylogenetic identification to metabolism can be challenging [160].

### Metaproteomics

Community proteomics (metaproteomics, Table 1) involves protein purification from an environmental sample, followed by mass spectrometry to characterize peptide masses and charges for comparison to a database of known protein sequences to identify proteins in the sample. Detailed reviews of metaproteomics and the many recently reported microbial metaproteomic studies across environments can be found elsewhere [161–163], but we highlight key strengths and weaknesses of this technique, in terms of identifying active microbial populations and metabolic processes. In contrast to mRNAs that have half-lives on the order of minutes, the average half-life of a protein within a bacterial cell is approximately 20 h, meaning that recovered proteins represent cells that were likely to have been active approximately within the last day [104, 164–166]. However, the addition of purified proteins to soil has shown that low levels of proteins can persist in the environment on timescales of at least months, with concentrations decreasing over time [167].

When linked to specific populations (e.g., via genomes in public databases and/or assembled metagenomic data [168–170]), protein-based detection can be used to identify not only which populations are viable but also their active metabolic pathways. Although metaproteomics shares some of the same drawbacks as mRNA-based approaches, in terms of implications for detecting living organisms, the sensitivity of any protein-based measurement is limited, compared to nucleic acid-based techniques, resulting in a reduced ability to detect low-abundance proteins [171]. For example, samples yielding gigabases of metagenomic sequence data corresponding to thousands of operational taxonomic units (OTUs) may only yield a few thousand unique protein identifications for the entire community [172]. In addition, because de novo protein sequencing is not yet possible for high-throughput protein detection, only proteins with exact sequence matches to the search database can be detected, highlighting the utility of a metagenomics-derived predicted protein database from the same sample.

### BONCAT for measuring translational activity

Bioorthogonal noncanonical amino acid tagging (BONCAT; Table 1), the in vivo incorporation of artificial amino acids into newly synthesized proteins by metabolically active cells, can be combined with “click chemistry” to measure the translational activity of microorganisms in environmental samples [173]. When the artificial amino acids are designed to have fluorescent properties, highly specific azide-alkyne click chemistry can be used to detect the fluorescent synthetic amino acids [174]. Designed to offer minimal interference with normal biological activity, this type of metabolic labeling has been used to detect the growth status of species and consortia in different environmental contexts. For example, following BONCAT activity-based cell sorting and 16S rRNA gene sequencing, consortia of Verrucomicrobia, other bacteria, and anaerobic methane-oxidizing (ANME) archaea were found to be active on aggregates from deep-sea methane seeps [175]. Although typically employed with a particular species or small consortium in mind, BONCAT can be coupled with rRNA-targeted FISH, fluorescence-activated cell sorting (FACS), multiple displacement amplification (MDA), and/or 16S rRNA gene sequencing to obtain phylogenetic information on the active fraction of the community [175, 176]. In principle, BONCAT is also compatible with metagenomic sequencing, though this has yet to be reported [175]. Relative to other techniques capable of measuring the activity of microbial consortia at the micron scale (as opposed to the scale of bulk samples, as required for most techniques described thus far), BONCAT is relatively inexpensive and requires less specialized equipment [175], rendering it a promising approach for future microbial ecological studies and an excellent candidate for further methods development across sample and organism types.

### Measuring genome replication rates from metagenomic data

As an expansion of the “peak-to-trough” method [177], an algorithm, iRep, has recently been developed to estimate genome replication rates from single-sample metagenomic data [178]. Both the peak-to-trough method and the iRep algorithm take advantage of a biological property of microbial genome replication: genomes are bi-directionally replicated from the origin of replication, resulting in a bias in the metagenomic sequencing coverage across the genomes from actively replicating populations. In actively replicating populations, more sequences are recovered from regions closer to the origin of replication, and greater coverage heterogeneity is observed across the genome. Inactive or slowly replicating populations have more homogeneous coverage throughout their genomes. Using genome sequences (which can include draft-quality population genomes recovered from metagenomes) and a single metagenome, iRep calculates an “index of replication” for each genome, which can be used to identify the actively replicating genomes in a sample [178].

### Membrane potential and respiratory activity

Membrane potential and respiratory activity are correlated because they both require a functional electron transport chain [22]. Seahorse XF kits and analyzers (Agilent Technologies, Santa Clara, CA), typically used on eukaryotic cells, can also be used to measure oxygen consumption rates and extracellular acidification rates as indicators of respiration and glycolysis, respectively, in bacteria [179]. In addition, carbocyanine dyes, such as bis-(1,3-dibutylbarbituric acid)trimethine oxonol (DiBAC_4_(3)), 3,3-diethyloxacarbocyanine iodide (DiOC_2_(3)), 3,3-dihexyloxacarbocyanine iodide (DiOC_6_(3)), 3,3′-dipropylthiadicarbocyanine iodide (DiSC_3_(5)), and rhodamine (RH123), have all been used to measure membrane potential and, by proxy, to evaluate the respiratory activity of cells [23]. Membrane stains routinely used to measure respiratory activity (e.g., DiBAC_4_(3)) are influenced by cell size [180], while the long staining times (4–24 h) required allow viability changes in the sample [181].

Alexa Fluor Hydrazide (AFH) has been used successfully to estimate the numbers of actively respiring bacteria in food and environmental samples. AFH dye is a photostable fluorescent molecule, which interacts with carbonyl groups in modified proteins of dead, dying, and aging cells, and it has been used as an alternative to DNA-binding dyes [182]. Saint-Ruf et al. [182] used a combination of culturing and cell sorting/flow cytometry to demonstrate that false-positive hits for AFH are very low for *E. coli* (only ~1.6% of the cells in an active cell culture were stained, and it is possible that at least some of the stained cells were actually dead or dying and therefore not false positives). Simultaneous staining with AFH and SYBR Green I or DiOC_2_(3) revealed that the majority of dead cells would not have been detected with the DNA-binding dyes alone. To the best of our knowledge, no attempts have been made to apply AFH to complex microbial communities.

### Isothermal microcalorimetry as a measure of heat flow

Isothermal microcalorimetry (IMC) is a non-destructive method that measures heat flow from biological processes produced by as few as 10,000–100,000 active bacterial cells [183]. There are a variety of types of microcalorimeters, but the basic format includes a reaction vessel containing the sample, which is connected to a heat sink via a thermopile that allows for the measurement of heat flow between the sample and the heat sink. The absence of pretreatment with exogenous dyes or other substances and the non-destructive nature of the technique make IMC a convenient complement to nearly any downstream analysis, including molecular studies. IMC has been used to study lakes [184, 185], marine sediments [186], and soils to investigate a variety of processes, including microbial activity, organic pollutant toxicity and degradation, and heavy metal (and metalloid) contamination [187]. One potential drawback of IMC is that the heat flow signal is a net sum of all biological, chemical, and physical processes taking place in an IMC ampoule, such that unknown phenomena may produce some of the heat measured, and simultaneous exothermic and endothermic processes could contribute to a misleading net signal [188].

## Assessing the abundance of viable spores in environmental samples

Spores or resting cysts—whether prokaryotic or eukaryotic in origin—pose a challenge for viability assessments, and, depending on the questions posed by a given study, researchers may or may not want to categorize viable spores as part of the living or active microbial community. Cultivation is still considered to be the gold standard for assessing viability for many spore-forming bacteria and other organisms because it is difficult to extract DNA from spores and distinguish between the presence of spore DNA and the presence of viable spores. The availability of alternatives to culture-based methods is critical for two primary reasons. First, there are regulatory and biosafety issues associated with culturing select agents, like the causative agent of anthrax, *Bacillus anthracis*. Second, reliance upon culturing assumes that the spore-forming microbes of interest can be cultivated using standard methods.

In general, the complex structure of spores by its very nature limits the utility of fluorescent dyes, such as acridine orange or 4,6-diamidino-2-phenylindole (DAPI), in determining spore viability when using epifluorescence microscopy or flow cytometry for total bacterial counts [189, 190]. Germinants such as amino acids or monosaccharides can be used to trigger activity in viable spores, which can then be detected using ATP bioluminescence and terbium dipicolinate fluorescence spectroscopy and microscopy [191]. However, these methods can substantially underestimate viable spore numbers in bacterial species that transition into a viable but non-cultivable state [26].

For a PMA assay to work on a spore, the PMA molecule must enter the core of a non-viable spore, passing through its different outer layers, in order to bind to DNA. Although PMA was reported to be a promising molecular technique for differentiating viable from non-viable spores [192], pretreatment of a spore suspension may be required to facilitate penetration of PMA up to the core in non-viable spores. Probst et al. used a 10 mM dithiothretiol (DTT) treatment (10 min incubation at 65 °C) before PMA treatment for detection of inactivated spores using fluorescence microscopy [193]. Further, a novel PMA-linked, FISH-based microscopic approach distinguished viable and non-viable spores of *Bacillus pumilus* SAFR-032, a bacterial contaminant common in clean rooms [191]. These studies were based on inactivated spore preparations only, and the broad applicability of these techniques to environmental samples is unknown. Future research should be conducted to determine if these promising approaches (PMA-fluorescence microscopy, PMA-FISH, and PMA followed by qPCR) could be used to detect viable spores of other bacterial and eukaryotic species, along with viable vegetative cells.

## Detecting viable (infective) viruses

There is a long-standing philosophical (and largely semantic) debate as to whether viruses should ever be considered to be alive [194]. A more practical consideration is whether or not viruses detected in a microbial community are infective, that is, still capable of infecting host cells. While the use of PMA to distinguish between infective and non-infective bacteriophage can be effective, this technique does not work on all viruses. For example, results from murine noroviruses showed that, while PMA could be used to distinguish between viable and non-viable virions, quantitative PCR following PMA treatment did not agree with culture-based results [66]. Recently, partial viral genomes (human cyclovirus 7078A and *Propionibacterium* phage P14.4) were reconstructed from clean room samples post-PMA treatment, potentially indicating the recovery of virions with intact capsids, and/or of viral genomes incorporated in viable bacterial cells [39]. The combined detection of both nucleic acids and capsid proteins (via proteomics or metaproteomics) may be more indicative of an infectious particle than nucleic acid detection alone [171], though viral proteomics seems to be better suited to identifying structural components of virions, rather than inferring viral infectivity [195, 196].

Although PMA and viral proteomics may be effective for screening for intact or infective viral particles in some cases, these techniques are not applicable to all viruses and are not currently recommended as a means of inferring viral infectivity in complex microbial communities. At this point, the most promising approach for detecting actively infecting viruses in microbial communities would be to mine bulk community metatranscriptomes and/or metaproteomes for viral transcripts and proteins. If these metabolites are detected across significant portions of a viral genome (as opposed to, for example, only the detection of structural capsid proteins in the metaproteome), then the signal most likely comes from active viral replication inside host cells. Of course, the detection of RNA viruses (i.e., viruses with RNA genomes) in a metatranscriptome is not necessarily indicative of viral activity.

## Distinguishing live from dead eukaryotic microbes

Eukaryotic microbes are vital members of most microbial communities as primary produces, grazers, and consumers. Some techniques outlined here can be applied to yeasts or protists with minimal or no modification, including the previously discussed LIVE/DEAD™ kits from ThermoFisher Scientific. A similar kit, the LIVE/DEAD™ Violet Viability/Vitality Kit (ThermoFisher L34958), relies on two dyes: CellTrace™ calcein violet, which indicates cell viability based on plasma membrane integrity, and LIVE/DEAD® Fixable Aqua fluorescent reactive dye, which measures esterase activity as a proxy for metabolism. As one environmental example of microbial eukaryotic community viability assessments, staining ballast samples with a combination of two vital, fluorescent stains [fluorescein diacetate (FDA) and 5-chloromethylfluorescein diacetate (CMFDA)], followed by epifluorescence microscopy, allowed for the direct enumeration of live protists between 10 and 50 μm in diameter [197].

Photosynthetic protists, whether algal or containing photosynthetic endosymbionts, present a special problem for dye-based assays because chlorophyll is autofluorescent. This makes propidium iodide staining unsuitable for chlorophyll-bearing organisms, including cyanobacteria, because the fluorescent signal of the stain overlaps with the autofluorescence of chlorophyll. Thus, Sato and colleagues developed a technique based on using SYTOX Green, a dye that penetrates compromised cell membranes, in combination with the autofluorescence of chlorophyll, to distinguish living from dead cells [198]. This technique has been used for microalgae (chlorophytes) as well [199]. Of course, this method only works if chlorophyll is still present.

## Conclusions

A variety of techniques exist to assess the relative proportions of living and dead microorganisms in natural environments. It will be particularly useful to integrate viability assays with large-scale, culture-independent, sequencing-based studies of microbial communities. Some assays, such as PMA and RNA analyses, lend themselves well to such studies, providing insight into the specific populations that are living. When compared to controls such as PMA-untreated samples or DNA sequences, respectively, these analyses can also uncover populations likely to be dead. For researchers seeking to identify living and dead populations as a small component of a more comprehensive microbial ecological study, PMA treatment followed by sequencing PCR amplicons or RNA analyses are likely to be broadly applicable (and less likely to require extensive trouble-shooting), though methods optimization may be inevitable. PMA has already been applied to at least one metagenomic study, and further development and application of this technique will improve our ability to assess the metabolic potential of active microbial populations, particularly those at low relative abundance. Another encouraging technique, demonstrated for specific microbial populations and consortia and in its early stages of development for more complex microbial communities, is BONCAT, which relies on the incorporation of labeled amino acids into proteins in actively translating microbial cells during an incubation. This minimally invasive procedure is compatible with many downstream applications, including DNA sequencing, which may not be possible for techniques that label the nucleic acids themselves.

For researchers seeking high-throughput counts of living and dead microorganisms without the resolution of specific populations, dye treatment followed by flow cytometry or PMA treatment followed by qPCR may be appropriate, depending on the environment. Although flow cytometry has proven to be a relatively robust counting technique in marine and freshwater systems, extensive methods development may be required to generate meaningful results (both from live/dead assessments and from bulk counts) in other systems, due to background autofluorescence and non-specific dye binding. For optimal interpretation of flow cytometry results, we encourage reporting representative results (e.g., the side scatter vs. fluorescence intensity graph, or equivalent, with an indication of the counted region) from flow cytometry standards, samples, and blank controls, particularly from non-aqueous systems. Most systems should be amenable to qPCR (and digital qPCR) for quantifying microbial biomass via 16S rRNA gene or other conserved gene amplification, but DNA extraction efficiency, gene copy number, and primer bias can all affect the absolute counts. For live/dead qPCR quantification, qPCR can be performed after PMA treatment, which has now been successfully applied to a variety of environments.

Still, further methods development is necessary to evaluate the applicability of any live/dead technique to a new organism, sample type, and/or community. For viability assays applied to any complex community, we encourage careful selection and validation of the assay, based on the physical and chemical composition of the sample and the expected diversity of microorganisms and metabolic states, along with the use of appropriate controls to assist with interpretation.

This piece began on a philosophical note, with the provocative title “Schrödinger’s microbes,” suggesting that organisms can be in two states at the same time. While Schrödinger’s cat was meant as a thought experiment, the very idea of a microbe being both live and dead simultaneously as an artifact of observational techniques lends itself to experimental proof. The assumption of membrane integrity as a biomarker for life is breached every time electroporation is used, a method in which membrane integrity is temporarily compromised as a result of electric shock, in order to allow the passage of DNA from the medium into the cells. Thus, we hypothesized that for some period of time after electroporation, a membrane integrity test of life would result in a false negative.

**Fig. 7:**
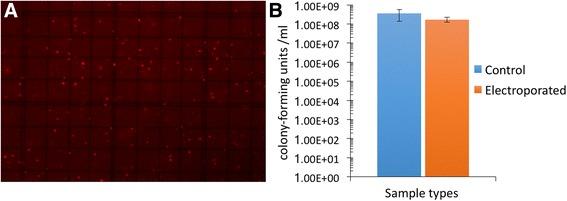
A transiently “dead” microbe. Competent *E. coli* (NEB5α cells competent cells, cat # c2987, New England Biolabs, Ipswitch, MA, USA) were thawed on ice. For a control sample, 2 μL of cells was added to 98 μL LB culture medium, 100 μL propidium iodide added, and the mixture allowed to stain for 5 min at room temperature. The experimental *E. coli* NEB5α (25 μL) was added to an electroporation cuvette previously cooled to 5 °C and electroporated at 2500 V twice. The cells were then diluted in LB and stained as with the control cells. **a** Fluorescence microscopy showing that almost all cells stained positive for propidium iodide treatment. **b** Colony-forming units showing no significant difference between controls and electroporated samples

To test this hypothesis, competent *E. coli* cells were stained with propidium iodide and then electroporated. The total cell concentration before electroporation was 1.74 (±0.226) × 10^9^ cells mL^−1^ as revealed by cell counts using a hemocytometer under a Zeiss Asioskope microscope. After electroporation and staining with propidium iodide (Fig. 7a), the cell count was 1.6 × 10^9^ cells mL^−1^, which translates to almost 92% “dead” cells. When cell viability was assessed by serial dilution and plating, non-electroporated controls showed 3.5 (±2.12) × 10^8^ colony-forming units mL^−1^, while electroporated samples showed 1.7 (±0.495) × 10^8^ cells mL^−1^ (Fig. 7b). This quick experiment demonstrates that although the staining procedure with propidium iodide immediately after electroporation suggested that over 90% of *E. coli* were “dead,” viability assessment through serial dilution and plating showed no significant difference between controls and electroporated samples. Similarly, Krüger et al. [200] showed that when they transiently altered the metabolic state of the bacterium *Campylobacter* by abolishing the proton-motive force or by inhibiting active efflux, the resulting colony-forming units were the same as the untreated (control) samples, but enhanced entry of ethidium bromide into the bacteria was observed, which should be indicative of dead cells.

As these experiments so clearly demonstrate, determining death in a microbe is a tricky undertaking that requires a balance of the practicality of the assay method and the appropriate interpretation of the results.

## Abbreviations

AFH: Alexa Fluor Hydrazide
ANME: Anaerobic methane-oxidizing
ATP: Adenosine 5′-triphosphate
BONCAT: Bioorthogonal non-canonical amino acid tagging
DAPI: 4′,6-Diamidino-2-phenylindole
DGGE: Denaturing gradient gel electrophoresis
DMSO: Dimethyl sulfoxide
DNA: Deoxyribonucleic acid
DTT: Dithiothretiol
EDTA: Ethylenediamine tetraacetic acid
EMA: Ethidium monoazide
FACS: Fluorescence-activated cell sorting
FISH: Fluorescence in situ hybridization
IMC: Isothermal microcalorimetry
LAMP: Loop-mediated isothermal amplification
LED: Light-emitting diode
MDA: Multiple displacement amplification
mRNA: Messenger ribonucleic acid
MVT: Molecular viability test
OTU: Operational taxonomic unit
PCR: Polymerase chain reaction
PI: Propidium iodide
PMA: Propidium monoazide
pre-RNA: Precursor ribonucleic acid
qPCR: Quantitative polymerase chain reaction
RLU: Relative light units
RNA: Ribonucleic acid
rRNA: Ribosomal ribonucleic acid
RT-PCR: Reverse transcription polymerase chain reaction
SIP: Stable-isotope probing
